# Joint spatiotemporal evaluation of multiple healthcare resources: hospitals, hospital beds and physicians across 365 Chinese cities over 22 years

**DOI:** 10.3389/fpubh.2025.1642295

**Published:** 2025-09-12

**Authors:** Xin Qi, Mingyu Xie, Yaqian He, Xianteng Tang, Lingfeng Liao, Yaling Luo, Kaiwei Lin, Xiang Yan, Xiuli Wang, Yuanyuan Zhu, Zhangying Tang, Yumeng Zhang, Chao Song, Jay Pan

**Affiliations:** ^1^School of Public Health, Xi’an Jiaotong University, Xi’an, China; ^2^HEOA - West China Health & Medical Geography Group, West China School of Public Health and West China Fourth Hospital, Sichuan University, Chengdu, China; ^3^Chengdu Railway Prevention and Control Institute, China Railway Chengdu Bureau Group Corporation, Chengdu, China; ^4^Department of Geosciences, University of Arkansas, Fayetteville, AR, United States; ^5^Department of Geography, Indiana University Bloomington, Bloomington, IN, United States; ^6^State Key Laboratory of Oil and Gas Reservoir Geology and Exploitation, School of Geoscience and Technology, Southwest Petroleum University, Chengdu, China; ^7^College of Architecture and Environment, Institute of Urbanization Strategy and Architecture Research, Sichuan University, Chengdu, China; ^8^Institute for Healthy Cities and West China Research Centre for Rural Health Development, Chengdu, China; ^9^Health Promotion and Food Nutrition & Safety Key Laboratory of Sichuan Province, Chengdu, China

**Keywords:** regional healthcare resources, multiple indicators, joint spatiotemporal evaluation, spatiotemporal heterogeneity, healthy cities, SDG, China

## Abstract

**Background:**

Regional disparities in healthcare resource allocation across space and time present significant challenges to the global achievement of SDG 3, SDG 10, and SDG 11. To this end, we proposed a joint spatiotemporal evaluation framework to assess the synergistic efficiency of multiple healthcare resources.

**Methods:**

Using China as a case study, we analyzed data from 365 cities (2000–2021) on three key healthcare resource indicators: hospitals, hospital beds, and physicians. A composite healthcare resource score was constructed using the entropy weight method. We developed a three-dimensional joint spatiotemporal evaluation framework incorporating spatial Gini coefficient, emerging hotspot analysis, and Bayesian spatiotemporally varying coefficients (BSTVC) model with spatiotemporal variance partitioning index (STVPI) to evaluate spatiotemporal equity, agglomeration, and influencing factors. Individual indicators were evaluated to validate the framework’s robustness.

**Results:**

(i) Spatiotemporal description: The composite indicator, weighted by hospitals (25%), hospital beds (46%), and physicians (29%), showed only a modest increase from 2000 to 2021, with persistently lower values in western and northern regions. (ii) Common spatiotemporal equity: The spatial Gini coefficient for the composite indicator increased annually by 0.34%, mirroring trends in hospital beds (0.34%) and physicians (0.26%) but contrasting with hospitals (−0.32%). This suggested that declining equity was mainly driven by hospital beds and physicians, partially offset by the more balanced distribution of hospitals. (iii) Common spatiotemporal agglomeration: Hotspot intensity for the composite indicator was lower than that for hospitals but higher than that for hospital beds and physicians. Cold spots were more concentrated for the composite indicator than for any individual indicator, with less than 10% overlap across the three indicators, indicating weak regional synergy. (iv) Common spatiotemporal drivers: BSTVC and STVPI methods revealed consistent patterns of explainable percentages across four healthcare resource indicators, with population density (37.96%, 95% CI: 30.05–43.05%) and employed population density (31.63%, 30.69–33.83%) emerging as dominant common drivers, supporting unified and coordinated policy interventions.

**Discussion:**

We proposed a joint spatiotemporal evaluation framework to quantify both common and differentiated allocation patterns and driving factors across multiple healthcare resource indicators, highlighting the necessity for type-specific, temporally responsive, and spatially adaptive interventions to support dynamic monitoring and precise regulation of regional healthcare resource allocation globally.

## Graphical abstract

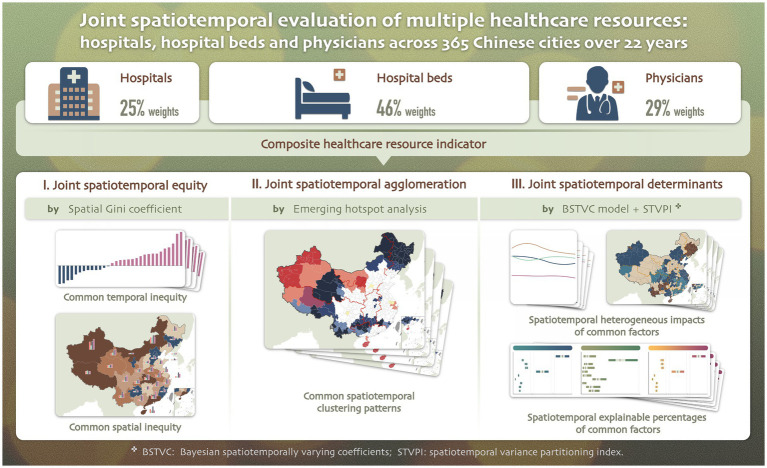

## 1 Introduction

The equitable allocation of regional healthcare resources constitutes a critical foundation for achieving universal health coverage (1, 2). However, the Universal Health Coverage Service Coverage Index (UHC SCI), proposed by the World Health Organization, has demonstrated a significant global decline since 2015, with regions exhibiting delayed progress in basic service coverage simultaneously manifesting substantial disparities in healthcare resource distribution (3). This pattern directly undermines the attainment of Sustainable Development Goals (SDGs) 3, 10, and 11, which respectively address good health and well-being, reduced inequalities, and sustainable cities and communities (4). Empirical evidence confirms a persistent spatial Matthew effect in healthcare resource allocation, characterized by concentrated abundant quality-care resources in developed areas and persistent scarcity in underdeveloped areas (5). Such spatial imbalances have progressively intensified over time (6), resulting in measurable spatiotemporal disparities in geospatial accessibility to healthcare services (7, 8). Previous studies on the allocation of healthcare resources and the delivery of healthcare services in China have revealed marked regional inequities and temporal variations in healthcare capacity (9). These findings indicate that overlooking spatial and temporal heterogeneity obscures local inequities and dynamic trends, highlighting the necessity for geographically and temporally tailored health-resource allocation policies (10, 11). A comprehensive spatiotemporal analysis framework therefore becomes imperative to analyze the spatiotemporal patterns of healthcare resource allocation, and ultimately enabling evidence-based policy formulation for optimizing resource allocation strategies (12, 13).

Previous studies have predominantly evaluated the healthcare resource allocation patterns and associated influencing factors from either spatial or temporal perspective (7, 14–16). These studies have largely relied upon an individual indicator, mainly focusing on the number of hospital beds, hospital density, or physician-to-population ratio to quantify resource allocation patterns and yield foundational insights for optimizing single-type healthcare resource. However, a couple of issues embedded in real-world health planning practices would enrich research in this area. First, the multi-dimensional nature of healthcare system, which compromises interdependent elements such as hospitals, physicians and hospital beds (17–20), necessitates integrated analysis to reveal the complex interactions among these elements. Physicians deliver essential clinical services, beds constitute the foundational infrastructure, and hospitals integrate these human and physical resources into a coordinated care delivery system (21–23). The optimal service delivery requires coordination of hospitals, physicians and hospital beds, as their interactive coordination fundamentally determines healthcare service efficiency (24). Above mentioned studies nevertheless persist in employing an individual indicator, disregarding the interactive dynamics between these core elements (25). Thus, current evaluations of healthcare resource allocation are inaccurate due to failure to consider interactions among multiple resources.

Second, the comprehensive spatiotemporal evaluation typically included three dimensions: spatiotemporal equity (26), spatiotemporal agglomeration (24), and spatiotemporal influencing factors (27–29). Theses dimensions collectively enabled spatiotemporal analysis of regional healthcare resource allocation, crucial for adaptive policymaking. Existing macro-level research, grounded in the theoretical framework of health determinants and using geographic administrative divisions as the unit of analysis (30), indicates that top-tier macro-scale natural environmental variables and social environmental factors jointly drive the spatiotemporal inequities in healthcare resource allocation (31–33). Existing research frequently neglected the systematic integration of these perspectives, resulting in fragmented understanding of resource pattern, potentially exacerbating resource misallocations (25, 34). Such methodological deficiencies would ultimately produce suboptimal regional strategies incapable of addressing real-world complexities, thereby undermining the achievement of SDGs.

In addition, the comprehensive evaluation approach, compared to single-indicator evaluation, improves analytical precision through systematic integration and weighting of multiple indicators, thereby fully capturing the real contribution and the relative importance of each type of resource. This approach reduces analytical biases associated with single-indicator evaluation while offering an integrated macro-level framework to evaluate spatial and temporal resource distribution patterns (35). When combined with spatiotemporal analytical dimensions, it provides an empirical basis for formulating evidence-based strategies to enhance allocation efficiency and equity (36). Crucially, integrating multi-dimensional spatiotemporal analyses of multiple indicators within the comprehensive evaluation framework not only validates the effectiveness of the composite indicator but also reveals intra-regional disparities that might otherwise be obscured. Such a spatiotemporal evaluation of healthcare resource allocation based on the synergy of multiple indicators, serves as a methodological advancement critical for addressing systemic health inequities and advancing progress toward SDGs (37).

China currently faces a notable challenge of healthcare resource maldistribution (12, 38, 39). The compounding effects of population aging (40, 41), accelerated urbanization (42), and policy-induced administrative boundary realignments (43) have exacerbated spatial inequities in healthcare resource allocation across urban China, thereby amplifying disparities in population health outcomes (44). This context necessitates a comprehensive spatiotemporal evaluation of multi-dimensional healthcare resource distribution patterns in Chinese cities, which would inform evidence-based intervention strategies to advance the implementation of the Healthy China 2030 Strategy (45, 46).

To address the challenges of proposing a joint evaluation framework toward multiple healthcare resource indicators from the spatiotemporal heterogeneity perspective, this study firstly constructed a composite healthcare resource indicator integrating hospitals, hospital beds, and physicians across 365 Chinese cities (2000–2021) using the entropy weight method. Then, a three-stage joint spatiotemporal evaluation framework was developed: (i) spatial Gini coefficient analysis to assess common spatiotemporal equity dynamics, (ii) emerging hotspot detection to capture common spatiotemporal agglomeration patterns, and (iii) Bayesian spatiotemporally varying coefficients (BSTVC) model with spatiotemporal variance partitioning index (STVPI) to identify common socioeconomic and environmental determinants and quantify their spatiotemporal explainable percentages. Our approach is expected to advance SDG-oriented resource optimization by enabling spatially and temporally adaptive policy design, with applications extending to global contexts facing similar challenges in balancing equitable distribution and targeted health planning.

## 2 Data and methods

### 2.1 Data

This study utilized panel data spanning 2001 to 2021 from 365 Chinese cities, including three healthcare resource indicators, namely hospitals, hospital beds, and licensed (assistant) physicians, and 34 socioeconomic and environmental influencing factors (24, 27), which were provided in Supplementary Table S1. Healthcare resource and socioeconomic data derived from China City Statistical Yearbook (2000–2021),^1^ while environmental metrics sourced from validated remote sensing platforms. For example, SO_2_, PM_2.5_, and normalized difference vegetation index (NDVI) were obtained from the Giovanni platform,^2^ PM_1_ and PM_10_ were obtained from the Zenodo platform,^3^ and nighttime light data were obtained from the Figshare platform (47).

We employed the Healthcare Resource Density Index (HRDI) as the dependent variable for single indicators, mitigating single-metric bias inherent in population- or area-exclusive denominators (12). The HRDI is defined as:


(1) $$\mathrm{HRD}I_{\mathrm{ijt}}=\sqrt{\left(y_{\mathrm{ijt}}/P_{\mathrm{it}})(y_{\mathrm{ijt}}/A_{\mathrm{it}}\right)}$$

where *i*, *j* and *t* refer to city *i*, healthcare indicator *j* and year *t*, *y_ijt_* refers to the quantity of city *i’*s healthcare resource *j* in year *t*, *P_it_* refers to the number of city *i’*s total population in year *t*, and *A_it_* is city *i’*s land area in year *t*.

The composite healthcare resource score, derived via the entropy weighting method (48), serves as the main dependent variable. This objective weighting technique applies information entropy principles to calculate indicator-specific weights through Equation (1) based on dispersion magnitude, thereby eliminating subjective bias and quantifying the relative importance of healthcare resource indicators.

The independent variables included socioeconomic and environmental factors related to population and economy, industry and employment, education and human resources, and environment and infrastructure. This study employed variance inflation factor (VIF) and node purity indicator for variable selection. We set a VIF threshold of 5 to identify variables with lower multicollinearity, and used node purity indicator to quantify explanatory significance through complex nonlinear relationships and interaction effects.

### 2.2 Methods

#### 2.2.1 Joint spatiotemporal evaluation framework

**Figure 1:**
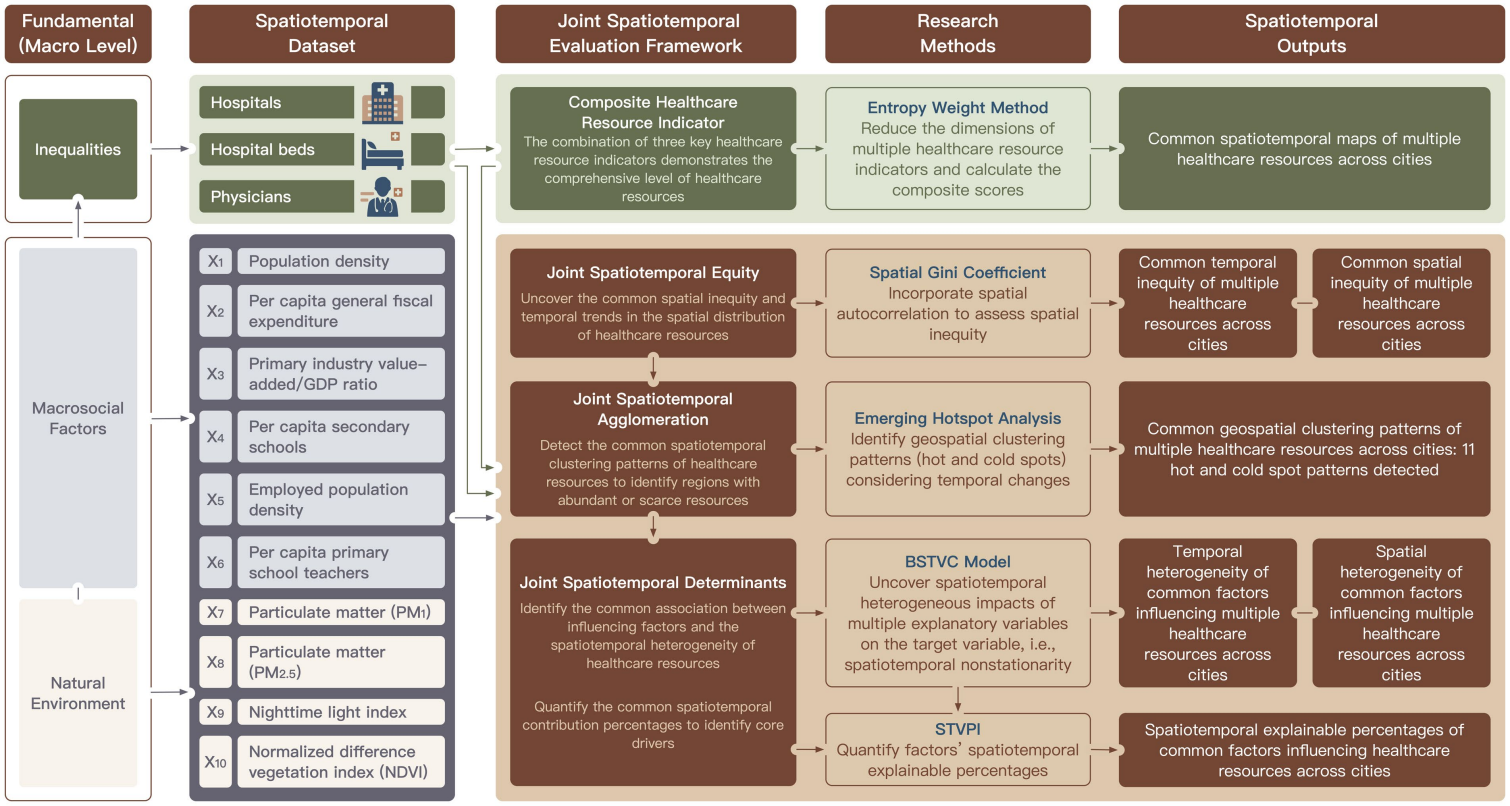
Joint spatiotemporal evaluation framework for multiple regional healthcare resource indicators, integrating hospitals, hospital beds, and physicians across 365 Chinese cities over a 22-year period. In the first column of this framework, we present the relevant theoretical model “*Public health framework for use in health impact assessment and health profiling*” (30). This study addresses only the macro-scale, excluding the meso, micro, individual, and population levels.

This study established a multi-dimensional spatiotemporal evaluation framework for city healthcare resource allocation in China, utilizing spatiotemporal panel data from Chinese cities spanning all 22 years from 2000 to 2021. Guided by health determinants theory, this study focused on macro-scale social and natural environmental factors that shaped spatiotemporal inequities in healthcare resource allocation. Figure 1 details the analytical workflow: (i) computation of composite scores via entropy weighting, (ii) quantification of the common equity dynamics through spatial Gini analysis, (iii) identification of common clustered spatial patterns and temporally evolving priority regions via emerging hotspot detection, (iv) revelation of the common spatiotemporal heterogeneous impacts of influencing factors using the BSTVC model and (v) quantification of spatiotemporal contributions of explanatory factors via STVPI. Steps ii to v underwent parallel implementation for single-indicator evaluation.

#### 2.2.2 Spatiotemporal equity evaluation

The spatial Gini coefficient quantifies geographic disparities in healthcare resource distribution by incorporating inter-jurisdictional allocation dynamics, thereby capturing spatial heterogeneity absent in conventional Gini metrics (26). It is a powerful tool for understanding the inequities in resource distribution across different regions. Values range theoretically from 0 to 1, with lower values indicating spatially balanced allocations and higher values reflecting concentrated distributions (49). This coefficient has been widely applied in various fields to assess the spatial equity of resource allocation (50). This study computed the spatial Gini coefficient using the *lctool* package in R 4.4.0 (51), which is a reliable and efficient tool for spatial analysis.

#### 2.2.3 Spatiotemporal agglomeration evaluation

Emerging hotspot analysis applies the Getis-Ord Gi* statistic to quantify spatiotemporal clustering of healthcare resource allocation efficiency. This method has been widely utilized in spatial data analysis to detect crucial patterns of clustering across different regions (52, 53). Statistical outputs (*Z*-scores and *p*-values) identify notable spatial autocorrelation patterns, which helps researchers and policymakers understand the distribution dynamics of healthcare resources. The hotspots manifest as high-value units surrounded by high-value neighbors, while coldspots exhibit low-value units embedded in low-value surroundings (54), thereby providing a clear visualization of areas with concentrated resources and areas in need of improvement.

#### 2.2.4 Spatiotemporal drivers evaluation

The BSTVC model is a class of local spatiotemporal regression based on Bayesian statistics (55–57). A key advantage of this model is its ability to use a single “full-map” framework to uniformly capture spatiotemporal variations across all local regression coefficients (58). This enables precise identification of the spatiotemporal heterogeneous impacts of explanatory variables on the target variable, thereby revealing spatiotemporal nonstationarity. The BSTVC model offers a powerful tool for uncovering the complex spatiotemporal dynamics and influencing mechanisms affecting the target variable (55). The BSTVC model can be formulated as follows:


(2) $$\log(y_{\mathrm{it}})=\sum_{k=1}^{K}\mu_{\mathrm{ik}}X_{\mathrm{itk}}+\sum_{k=1}^{K}\gamma_{\mathrm{tk}}X_{\mathrm{itk}}+\varepsilon_{\mathrm{it}}$$

(3) $$\begin{array}{l} \mu_{\mathrm{ik}}|\mu_{-\mathrm{ik}}∼N({\bar{\mu }}_{\omega_{i},k},\frac{\sigma_{\mathrm{\mu k}}^{2}}{n_{\omega_{i},k}}),\gamma_{\mathrm{tk}}|\gamma_{t-1,k},\gamma_{t-2,k}\\ \sim N(2\gamma_{t-1,k}+\gamma_{t-2,k},\sigma_{\mathrm{\gamma k}}^{2}),\varepsilon_{\mathrm{it}}\sim N(0,\sigma_{\varepsilon }^{2}) \end{array}$$

where *y_it_* refers to the number of healthcare resource in city *i* at time *t*. The independent variables *X* constitute a *K*-dimensional vector of socioeconomic and environmental covariates exhibiting spatiotemporal heterogeneity, which collectively influence the dependent variable *y*. Local parameters *μ_ik_* and *γ_ik_* refer to the *k*-th *X*’s space-coefficients (SCs) and time-coefficients (TCs). *ε_it_* represent the modeling residuals. We applied a log transformation since its distribution was skewed. This achieved near-normality, met model assumptions, and improved reliability, fit, and predictive accuracy. It also dampened outliers and more robustly captured underlying relationships and overall data patterns.

The STVPI is designed to quantify and compare the explainable percentages of different spatiotemporal heterogeneous impacts (59). By calculating spatiotemporal contributions, it clarifies key driving factors (55, 60). Based on BSTVC modeling results, the STVPI further breaks down the total variance of each explanatory variable into two independent components: temporal nonstationary random effects and spatial nonstationary random effects. This decomposition helps reveal the sources of spatiotemporal variations in the data (55, 60). Based on the BSTVC model, the STVPI can be represented as follows:


(4) $$\rho_{k}=\frac{\sigma_{\mathrm{\mu k}}+\sigma_{\mathrm{\gamma k}}}{\sum_{k=1}^{K}(\sigma_{\mathrm{\mu k}}+\sigma_{\mathrm{\gamma k}})+\sigma_{\varepsilon }}\times 100\%$$

where *ρ_k_* is the spatiotemporal explanatory proportion of the k-th explanatory factor, with a range of [0, 100]. *σ_μk_* and *σ_γk_* represent the variance component of the k-th explanatory factor attributable to spatial non-stationarity and temporal non-stationarity, respectively. *σ_ε_* represents the residual variance which captures the unexplained stochastic variation. This study performed the BSTVC model according to Equations (2, 3) and STVPI analysis according to Equation (4) via R package *BSTVC* in R 4.4.0 (61).

## 3 Results

### 3.1 Descriptive mapping of multiple healthcare resources

**Figure 2:**
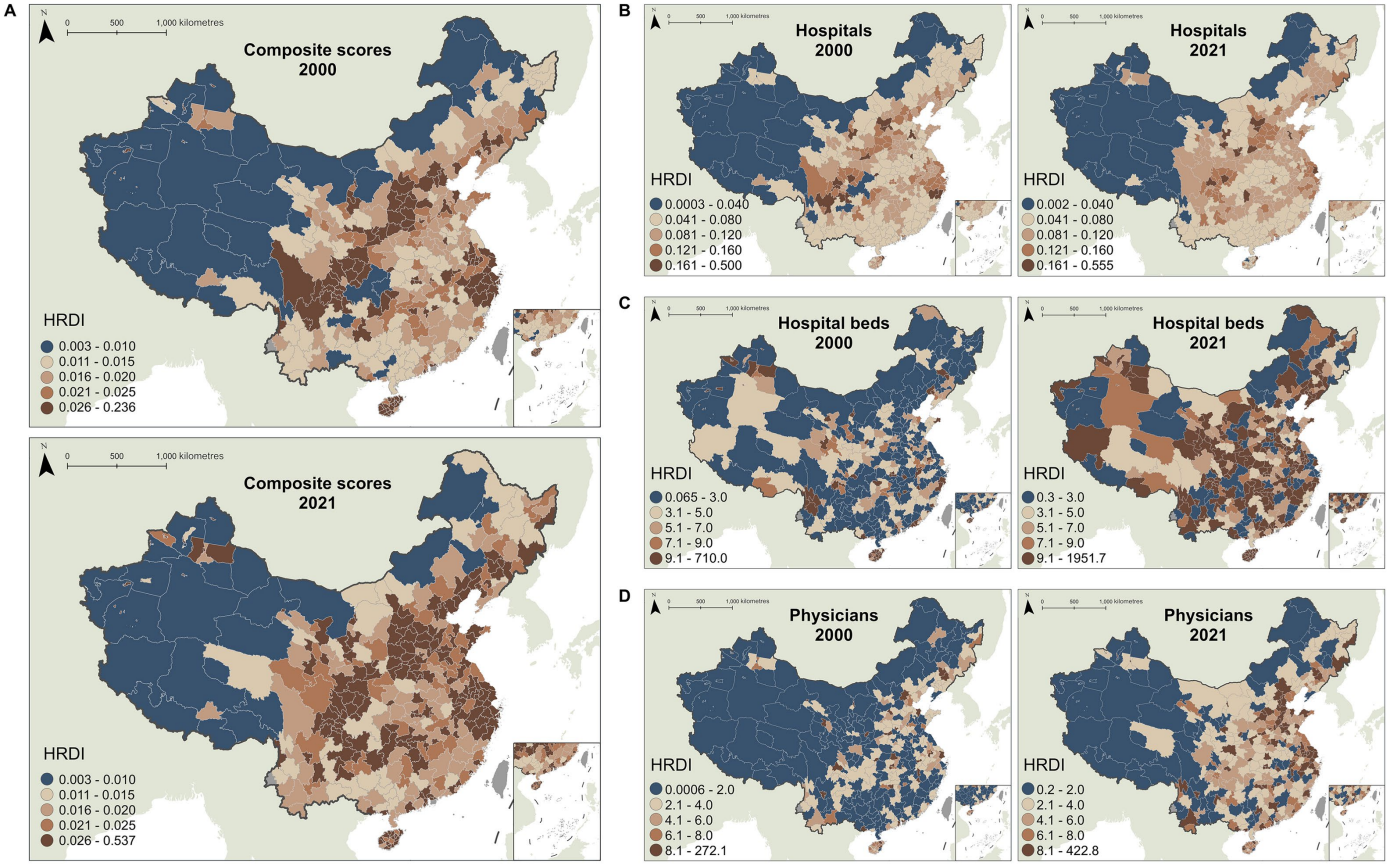
Spatial distribution of the Healthcare Resource Density Index (HRDI) for the **(A)** composite scores, **(B)** hospitals, **(C)** hospital beds, and **(D)** physicians across Chinese cities in 2000 and 2021.

The composite index of healthcare resources calculated through entropy weighting of hospitals, beds, and physicians, with respective weights of 0.25, 0.46, and 0.29, indicating that hospital beds’ main influence on resource allocation. The spatiotemporal analysis revealed that, the growth of city healthcare resources during the study period was relatively small and exhibited an inequitable pattern, characterized by lower resource levels in the west compared to the east, and in the north compared to the south. The composite score exhibited minimal average annual growth, demonstrating a modest increasing trend since 2007 with annual growth rates not exceeding 5%. Hospital density displayed a U-shaped trajectory, transitioning from initial decline to gradual increase after 2013, with growth rates remaining below 3%. Bed and physician densities followed similar growth patterns, both showing sustained increases since 2004 and reaching maximum annual growth rates of 10% (Figure 2).

### 3.2 Common spatiotemporal equity of multiple healthcare resources

**Figure 3:**
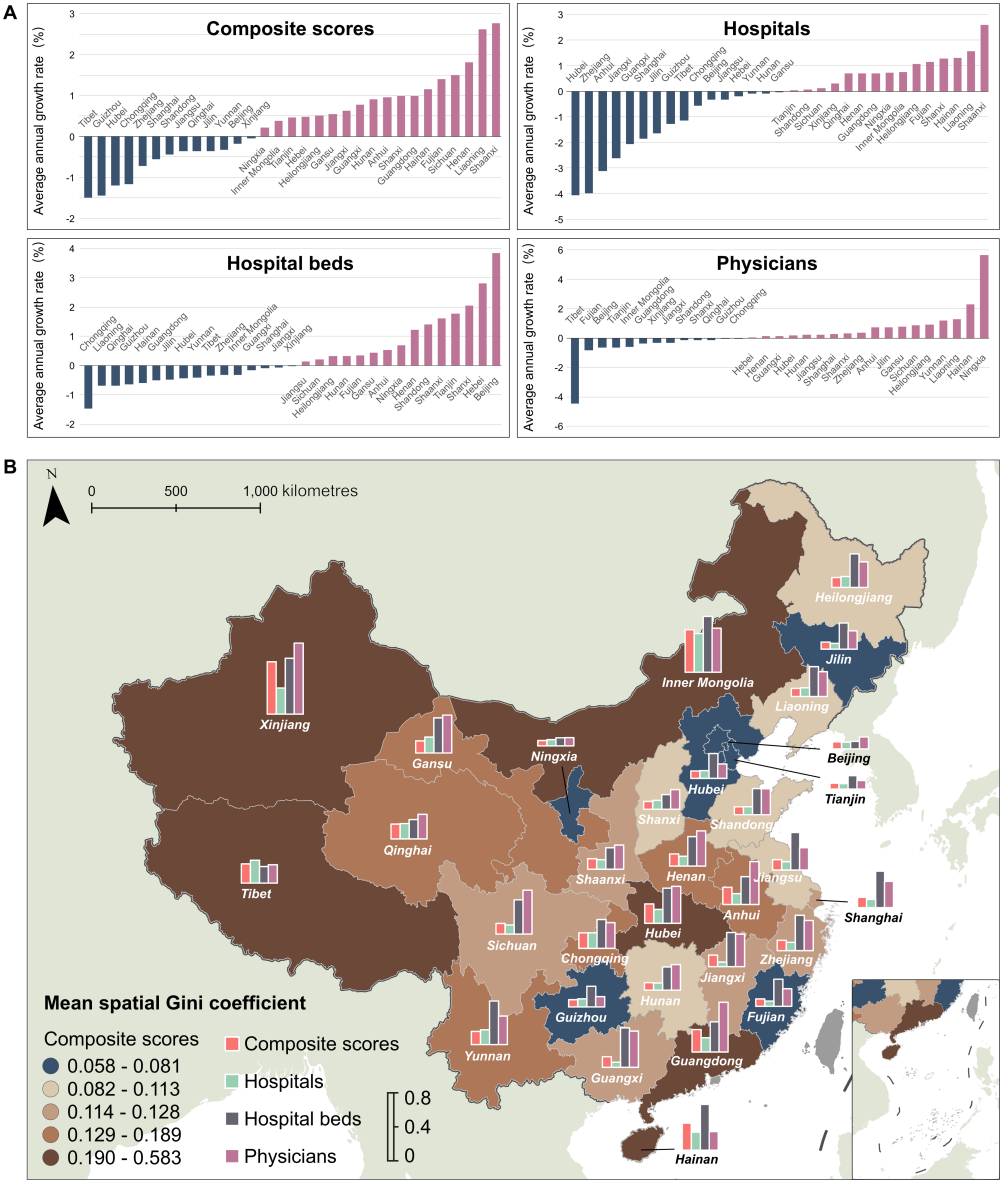
Spatiotemporal equity of multiple healthcare resource indicators: **(A)** average annual change in spatial Gini coefficients; **(B)** spatial distribution of the mean spatial Gini coefficient within cities at the provincial level.

Figure 3A presents the average annual changes in spatial Gini coefficients for the composite index and three single indicators during the study period. The composite index showed an overall decline in spatial equity, with an average annual increase of 0.34% in its spatial Gini coefficient. Regional analysis revealed that 18 of 31 provinces experienced reduced equity (maximum increase: 2.77% in spatial Gini coefficient), while the remaining 13 provinces showed improved equity (maximum decrease: 1.49%). These trends correlated with declining equity in bed indicator (0.34% annual increase) and physician indicator (0.26% annual increase).

Analysis of single indicators revealed distinct spatial equity patterns: bed indicator showed equity deterioration in 15 provinces (maximum annual increase: 3.86% in spatial Gini coefficient) versus improvement in 16 provinces (maximum decrease: 1.46%), while physician indicator exhibited declines in 18 provinces (maximum increase: 5.66%) and gains in 13 provinces (maximum reduction: 4.44%). In contrast, hospital indicator demonstrated nationwide improvement with an annual spatial Gini coefficient decrease of 0.32%. As shown in Figure 3B and listed in Supplementary Table S2, there were strong concordance between composite index equity and bed and physician equity. Regions with lower composite index equity consistently displayed poorer bed and physician equity, whereas high-equity regions exhibited smaller inter-indicator disparities.

### 3.3 Common spatiotemporal agglomeration of multiple healthcare resources

**Figure 4:**
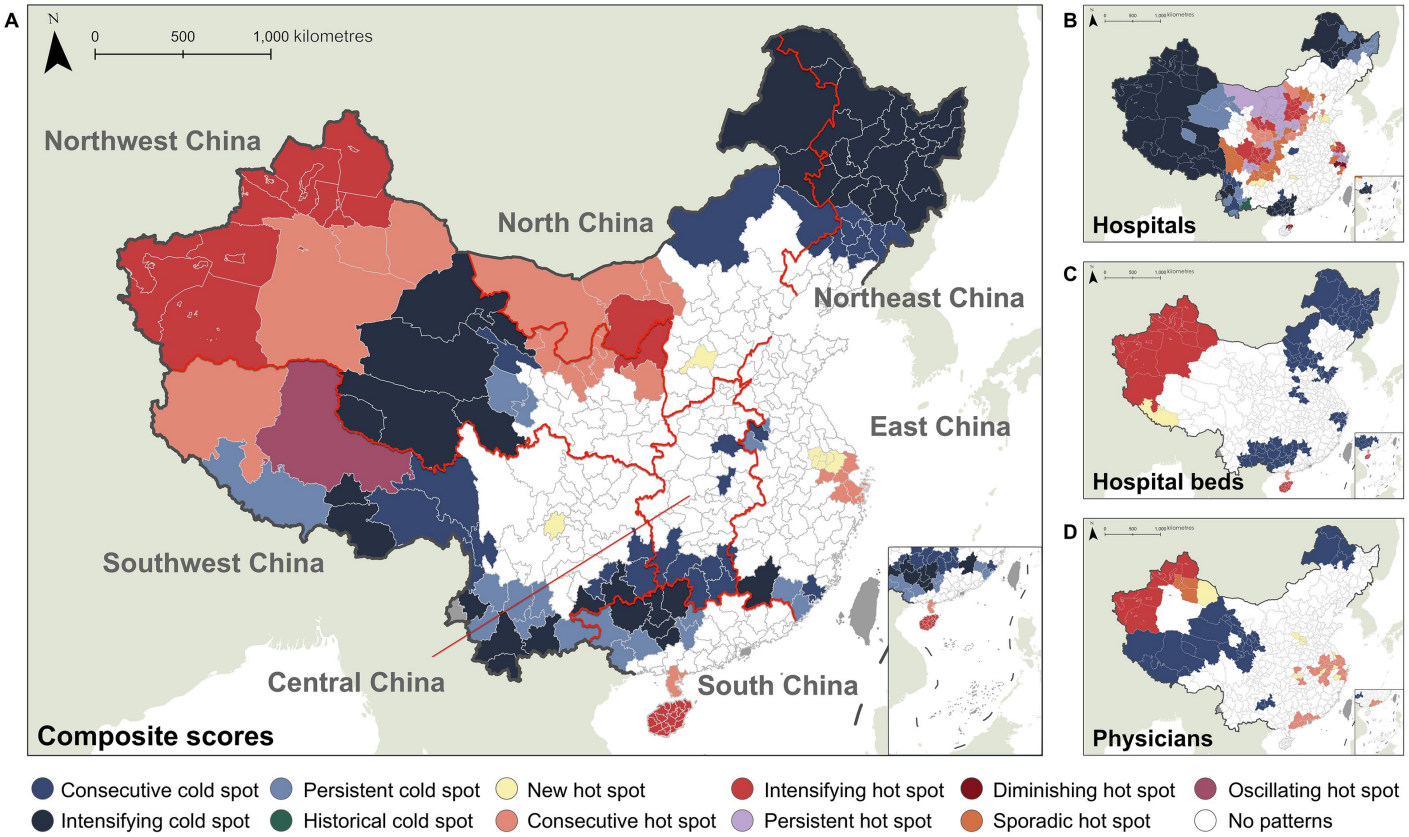
Spatiotemporal agglomeration patterns of healthcare resources in Chinese cities: hotspot and coldspot distributions for the **(A)** composite scores, **(B)** hospitals, **(C)** hospital beds, and **(D)** physicians.

Figure 4 shows spatiotemporal hotspot and coldspot distributions of city healthcare resources in China during the study period, with hotspots representing statistically significant resource-abundant clusters and coldspots indicating resource-deficient clusters. This study identified eleven distinct spatiotemporal agglomeration patterns. Supplementary Table S3 lists the detailed numbers of spatiotemporal agglomeration patterns for composite index and three single indicators. Supplementary Text S1 clarifies the specific meanings of these hotspots and coldspots.

The city-level composite index revealed that northwest China and coastal East China were predominantly characterized by intensifying and consecutive hotspots, while northeast and southwest regions manifested primarily as intensifying, consecutive, and persistent coldspots. Above patterns were substantially consistent with single indicator analysis. Nationally, 72 hotspot cities (19.73%) and 90 coldspot cities (24.66%) were identified. Quantitatively, the comparison of hotspot distributions across indicators revealed distinct patterns. The composite score exhibited fewer hotspots than hospital indicator (*n* = 105), but more than physician (*n* = 68) and bed indicator (*n* = 45). Conversely, coldspot counts for the composite score significantly exceeded all single indicators (hospital = 76, bed = 82, physician = 29). Structurally, 91.67% of the composite score hotspots cities overlapped with hotspots in at least one single indicator (hospital, bed, or physician), while 70.0% of the composite score coldspots cities coincided with coldspots in at least one specific resource indicator (hospital, bed, or physician). The observed spatiotemporal polarization of composite index demonstrated an intensifying Matthew effect in China’s city healthcare resource allocation, where the disparities between resource-rich and resource-deficient regions had progressively widened over time, with disadvantaged areas facing increasing challenges in closing the gap. The composite index exhibited strong representativeness of all three single indicators, as evidenced by both hotspot and coldspot quantification and structural alignment.

### 3.4 Common spatiotemporal drivers of multiple healthcare resources

#### 3.4.1 Common influencing factor selection

Following VIF screening, we retained 15 socioeconomic and 4 environmental factors from an initial set of 34 factors, as listed in Supplementary Table S4. The subsequent node purity indicator ranking identified the top 10 low-collinearity and high-importance variables, including population density (*X*₁), per capita general fiscal expenditure (*X*₂), primary industry value-added/GDP ratio (*X*₃), per capita secondary schools (*X*₄), employed population density (*X*₅), per capita primary school teachers (*X*₆), PM₁ (*X*₇), PM₂.₅ (*X*₈), nighttime light index (*X*₉), and NDVI (*X*₁₀).

Additionally, we employed Geographically Weighted Principal Component Analysis (GWPCA) to classify the time-varying and space-varying coefficients (TCs and SCs) derived from the BSTVC model, enabling the identification of dominant drivers of healthcare resource allocation and the characterization of localized spatiotemporal dynamics (62). The ten influencing factors were grouped into four principal components: (i) population and education (population density, per capita secondary schools, per capita primary school teachers), (ii) economic structure (per capita general fiscal expenditure, primary industry value-added/GDP ratio, employed population density), (iii) air quality (PM₁, PM₂.₅), and (iv) urbanization (nighttime light index, NDVI).

#### 3.4.2 Spatiotemporal heterogeneous impacts of influencing factors

**Figure 5:**
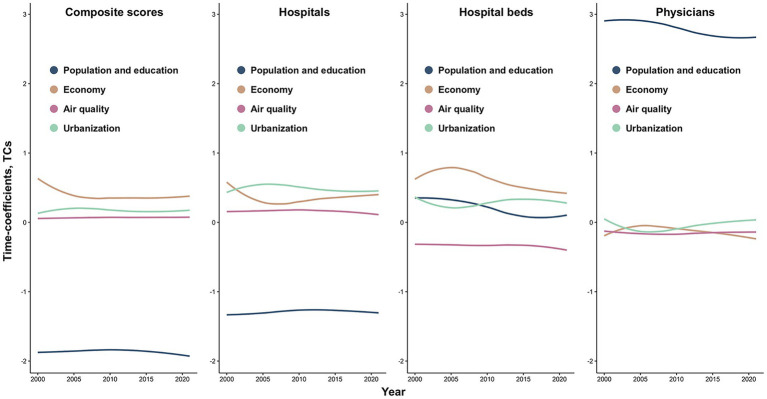
Temporally heterogeneous associations between healthcare resources and four categories of influencing factors from 2000 to 2021, based on time-varying coefficients (TCs) estimated using the BSTVC model. Factor categories include: population and education (population density, per capita secondary schools, per capita primary school teachers); economic structure (per capita fiscal expenditure, primary industry value-added/GDP ratio, employed population density); air quality (PM₁, PM₂.₅); and urbanization (nighttime light index, NDVI).

Figure 5 illustrates TC trends of the composite index and single indicators after dimensionality reduction. For the composite index, population and education exerted the strongest influence with increasing importance over time, while economic structure demonstrated diminishing effects, air quality and urbanization maintained stable impacts without notable temporal trends. Hospital indicator exhibited TC dynamics consistent with the composite index. Conversely, bed indicator showed divergent trajectories: air quality gained incremental importance while other factors declined. Physician indicator revealed declining influence of population and education alongside growing economic structure effects, with remaining factors fluctuating without statistically crucial trends. Collectively, population and education and economic structure emerged as temporally dynamic priority factors, whereas air quality and urbanization impacts remained comparatively stable longitudinally.

**Figure 6:**
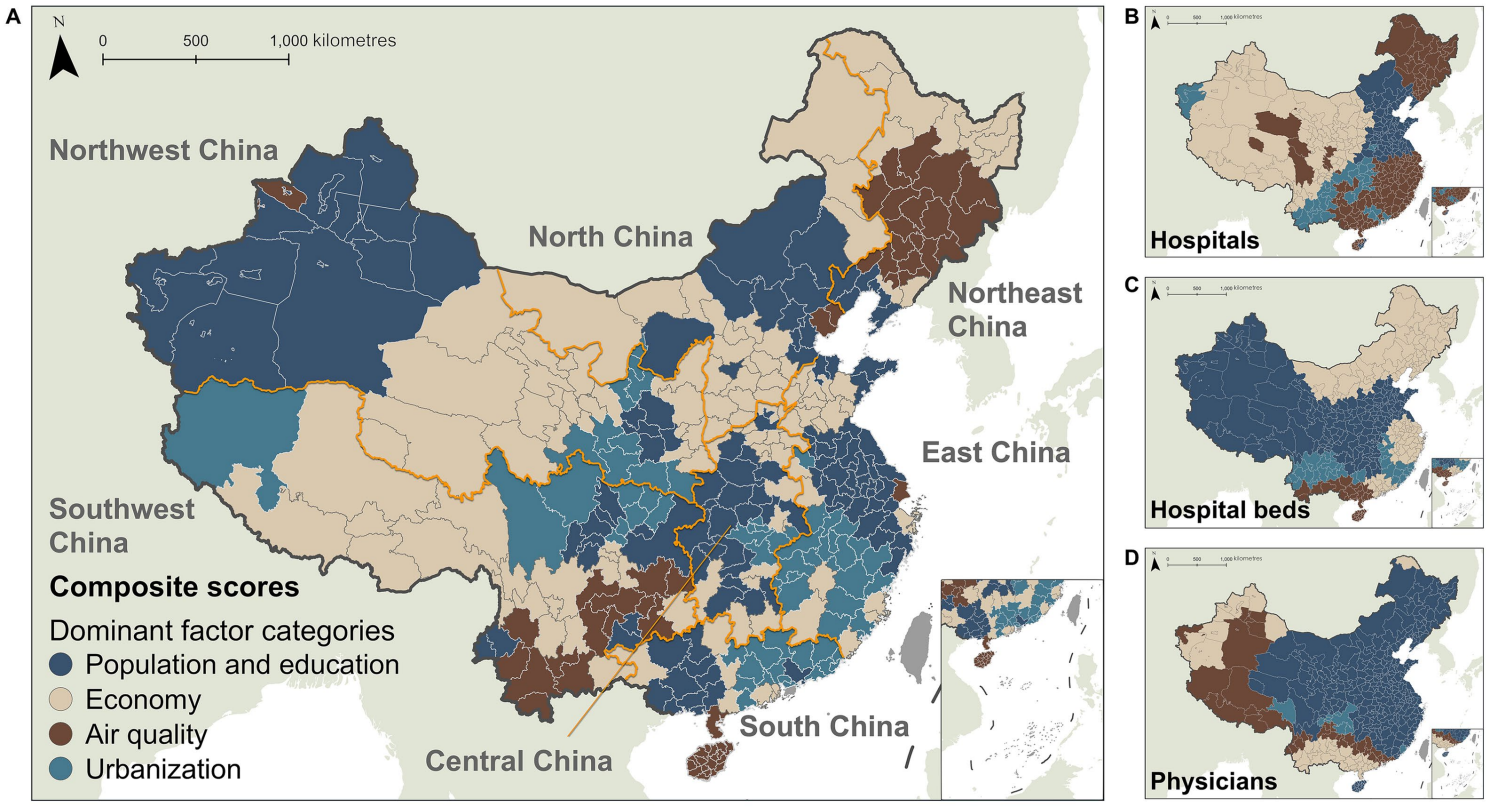
Spatially heterogeneous associations between healthcare resources and four categories of influencing factors, based on space-varying coefficients (SCs) estimated by the BSTVC model for **(A)** the composite scores, **(B)** hospitals, **(C)** hospital beds, and **(D)** physicians. The four factor categories include: population and education (population density, per capita secondary schools, per capita primary school teachers); economic structure (per capita fiscal expenditure, primary industry value-added/GDP ratio, employed population density); air quality (PM₁, PM₂.₅); and urbanization (nighttime light index, NDVI).

Figure 6 spatializes the dominant influencing factors for healthcare indicators derived via GWPCA across cities. The composite scores were predominantly influenced by population and education (125 cities, 34.25%) and economic structure (120 cities, 32.88%). Regarding specific single indicators, hospital indicator was primarily associated with air quality (149 cities, 40.82%) and economic structure (97 cities, 26.58%), while bed indicator was mainly determined by population and education (166 cities, 45.48%) and economic structure (122 cities, 33.43%). Physician indicator showed the strongest correlation with population and education (270 cities, 73.97%). Spatially, composite scores exhibited strong concordance with bed and physician indicators (population and education and economic structure), whereas hospitals contrastingly aligned with distinct factors (air quality and economic structure).

#### 3.4.3 Explanatory power of spatiotemporally heterogeneous drivers

**Figure 7:**
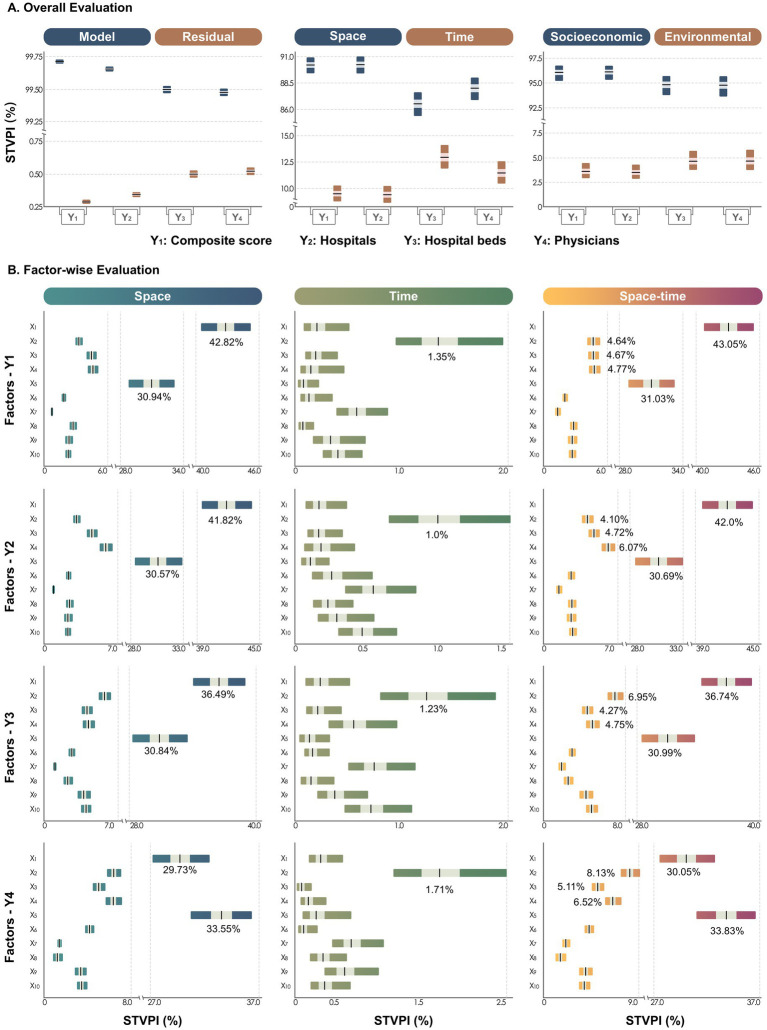
Spatiotemporal percentage contributions of explanatory factors (*X*_1_-*X*_10_) to healthcare resources across Chinese cities, quantified using the spatiotemporal variance partitioning index (STVPI). **(A)** Overall evaluation: Contribution percentages from the BSTVC model versus residuals; proportions attributed to spatial and temporal dimensions of shared influencing factors across all indicators; and the spatial and temporal heterogeneous associations between socioeconomic/environmental factors and healthcare resource indicators. **(B)** Factor-wise evaluation: Contribution percentages of each individual factor (*X*_1_-*X*_10_) to all healthcare resource indicators, assessed across spatial, temporal, and spatiotemporal dimensions. In two panels, the gradient color band represents the 95% confidence interval of each factor’s contribution, the white band represents the 50% confidence interval, and the black vertical line in the middle represents the mean.

Figure 7A shows the comprehensive evaluation of the relationship between the influencing factors and healthcare resources under spatiotemporal non-stationarity assumption. The Bayesian STVC model explained more than 99.48% (95% CI: 99.45–99.51%) of variance in composite and single indicators, with residual contributions less than 0.52% (95% CI: 0.49–0.55%), confirming model efficacy and result reliability. Spatial relationships accounted for 94.81% (95% CI: 93.67–95.70%) to 96.14% (95% CI: 95.35–96.76%) of explained variance, substantially exceeding temporal contributions (3.52% [95% CI: 2.90–4.31%] to 4.67% [95% CI: 3.78–5.81%]). Socioeconomic factors contributed 86.54% (95% CI: 85.38–87.62%) to 90.24% (95% CI: 89.41–91.02%) of variance, while environmental factors explained only 9.41% (95% CI: 8.65–10.24%) to 12.96% (95% CI: 11.89–14.11%), demonstrating socioeconomic determinants’ dominant role in healthcare allocation equity.

Figure 7B quantifies the contribution ratios of ten influencing factors across spatial, temporal, and spatiotemporal dimensions for composite and single indicators. Within each dimension, contribution ratios exhibited high consistency across indicators with minimal variation. Across different dimensions, the factors could be categorized into three tiers based on their contribution magnitude. Spatially, the primary contributors were population density (*X*₁) and employed population density (*X*₅), followed by per capita general fiscal expenditure (*X*₂), primary industry value-added/GDP ratio (*X*₃), and per capita secondary schools (*X*₄) as secondary factors, with per capita primary school teachers (*X*₆), PM₁ (*X*₇), PM₂.₅ (*X*₈), nighttime light index (*X*₉), and NDVI (*X*₁₀) constituting tertiary factors. Temporally, per capita general fiscal expenditure (*X*₂) emerged as the dominant factor, while PM₁ (*X*₇), nighttime light index (*X*₉), and NDVI (*X*₁₀) formed the secondary tier, with the remaining factors (*X*_1_, *X*_3_-*X*_6_, *X*_8_) showing relatively weaker contributions.

Spatiotemporally, the factor grouping pattern resembled the spatial dimension. Among primary contributors, population density (*X*_1_) demonstrated contribution ratios ranging from 30.05% (95% CI: 27.54–32.77%) to 43.05% (95% CI: 40.74–45.43%), with a mean of 37.96%, while employed population density (*X*₅) showed contributions between 30.69% (95% CI: 28.50–33.01%) and 33.83% (95% CI: 31.03–36.62%), with a mean of 31.63%. Secondary factors, including per capita general fiscal expenditure (*X*₂), primary industry value-added/GDP ratio (*X*₃), and per capita secondary schools (*X*₄), exhibited contribution ratios of 4.10% (95% CI: 3.61–4.70%) to 8.13% (95% CI: 7.29–9.11%). The remaining tertiary factors displayed contributions ranging from 1.31% (95% CI: 1.09–1.61%) to 4.67% (95% CI: 4.13–5.29%). Notably, population density and employed population density emerged as consistent spatiotemporal drivers across all four healthcare indicators, with cumulative contributions reaching 76.88%.

## 4 Discussion

This study advances a methodological triad for multidimensional spatiotemporal evaluation of city healthcare resources in China: (i) entropy-weighted composite index construction integrating hospitals, hospital beds, and physicians to examine macroscale equity dynamics, agglomeration patterns, and socioeconomic-environmental drivers; (ii) parallel spatiotemporal validation through individual indicator analyses, quantifying intra-regional disparities while verifying composite index robustness; (iii) dual-capacity analytical framework combining spatial Gini coefficients for strategic-level equity diagnostics with emerging hotspot detection for operational-scale resource surplus/deficit identification. The integrated approach reconciles spatial governance priorities by bridging systemic trend analysis (composite index) with precision targeting (individual indicators), enabling national policymakers to monitor allocation equity through aggregated metrics while empowering municipal authorities to implement context-specific interventions. This dual-scale architecture resolves the equity-precision paradox in resource governance, establishing a replicable protocol for SDG-aligned optimization of public infrastructure systems, with demonstrated applicability extending to education, ecology, and transportation networks through its modular analytical design.

### 4.1 Proposing a generic joint spatiotemporal evaluation framework for multiple healthcare resource indicators

This study’s joint spatiotemporal evaluation framework advanced multi-dimensional equity evaluation by simultaneously capturing localized asynchronous variations and systemic constraints of healthcare indicators while mapping macroscale allocation patterns. The results methodological diverged from single-indicator temporal analyses and nonspatial multi-dimensional studies (12, 24, 38), yet substantively aligned with United Nations statistical conclusions (3), confirming the critical role of spatiotemporal integration in comprehensive evaluation. Furthermore, the framework revealed the operational logic of allocation mechanisms through distribution pattern analytics, thereby providing robust theoretical support for policymakers to balance macro-level equilibrium with micro-level precise adjustments.

Specifically, this study systematically examined the spatiotemporal evolution and inter-regional coordination of China’s city healthcare resources from 2000 to 2021, revealing three critical findings. First, while aggregate healthcare resources measured via the composite index exhibited sustained increases, over half of Chinese regions concurrently experienced considerable equity declines, underscoring persistent systemic challenges in achieving equitable distribution. Second, the analysis revealed systemic interdependencies between composite and single indicators, highlighting the necessity for policymakers to sustain the stabilizing effects of incremental hospital equity improvements while prioritizing bed availability as a critical determinant of system-wide equity. Such a dual-strategy approach enabled targeted interventions to address allocation imbalances. Third, the framework identified polarized equity clusters, enabling dual-track policy responses: strategic replication of best practices from high-equity clusters and targeted interventions in low-equity areas exhibiting critical bottlenecks. These findings operationalized a multiscale governance model that integrated macroscale equity diagnostics with precision resource targeting, offering empirically validated pathways to reconcile aggregate growth with localized equity optimization in China’s healthcare system.

### 4.2 Prioritizing intervention strategies based on spatiotemporal disparities in regional healthcare resource allocation

This study advanced a multi-dimensional spatiotemporal evaluation framework to empirically identify critical healthcare allocation patterns and priority intervention targets. The composite index functioned as a macroscale diagnostic tool, exposing systemic allocation deficiencies and geospatial resource disparities, while parallel single indicator analyses revealed intra-regional inequities through comparative validation. This dual-scale analytical architecture provided policymakers with dual-capacity decision-support. The composite index dynamics guided macroscale intervention zoning, whereas single indicator evaluations served to identify short-board effects in specific resource categories at operational levels. Methodologically, the framework transcended descriptive spatiotemporal equity and spatiotemporal agglomeration analysis to a dynamic decision support system that bridged macro-equity monitoring with precision governance strategies, enabling simultaneous pursuit of system-wide equilibrium through strategic resource reallocation and localized optimization via context-specific short-board remediation.

This study utilized spatial Gini coefficient trajectories derived from composite index and single indicators to identify asynchronous dynamics in the equity of healthcare resource distribution across Chinese provinces, pinpointing regions with pronounced short-board effects. For example, Tibet achieved sustained improvement in equity through integrated policy interventions including centralized fiscal transfers (63), targeted aid programs (64), population redistribution policies (65), and the development of traditional healthcare system (66), thereby establishing a replicable governance model for sparsely populated and economically underdeveloped regions (67). In contrast, Sichuan experienced a decline in equity, primarily driven by resource concentration induced by population-economic gradients (68), and patients’ cross-regional health seeking behaviors (69). Above structural contradictions constrained equity improvements despite intervention efforts (70). These contrasting cases highlighted three critical policy imperatives, including enhancing the monitoring mechanism for the spatial impacts of population-economic gradients, optimizing allocation mechanisms to mitigate excessive resource polarization and implementing context-specific governance to address systemic bottlenecks, thereby identifying strategic priorities for achieving nationwide equitable healthcare distribution.

The spatiotemporal hotspot analysis identified 11 distinct agglomeration patterns of healthcare resource disparities across China, revealing systemic rather than isolated regional imbalances. Three geographically stratified paradigms were distinguished. Northeastern industrial bases exhibited resource depletion driven by economic contraction-induced fiscal austerity and population outflow-related service attrition (71). Yunnan-Guizhou plateau regions displayed suboptimal efficiency in central fiscal transfers due to dispersed settlements that impaired population-resource spatial matching. Yangtze River Delta regions manifested market-driven structural inequities where economic advancement coexisted with healthcare accessibility deficits despite resource abundance (72). These patterns collectively demonstrated the multiscale complexity of national healthcare imbalances, integrating macroeconomic volatility, mesoscale demographic-geographic mismatches, and market-mediated allocation mechanisms. These findings necessitate policy frameworks that reconcile macro-level equity objectives with micro-scale precision governance, addressing both fiscal constraints from economic cycles and population distribution impacts on allocation efficiency. The developed analytical model provides a methodological foundation for designing spatiotemporally adaptive interventions that balance systemic equity with context-specific effectiveness in complex resource allocation systems.

### 4.3 Identifying key socioeconomic and environmental drivers of spatiotemporal variations in multiple healthcare resources

The spatiotemporal analysis revealed distinct temporal dynamics and spatial patterns in healthcare resource allocation determinants. Temporally, economic development exerted immediate regulatory effects on resource distribution through rapid policy feedback mechanisms (73), while urbanization exhibited a three-phase U-shaped trajectory in hospital allocation, potentially reflecting municipal prioritization of economic growth over public service infrastructure development (44, 74). Spatially, the evaluation discrepancy between single indicators and composite index was attributed to spatial spillover effects that inter-city policy diffusion enhanced resource concentration (75), though regional heterogeneity in socioeconomic contexts and policy enforcement shaped final composite outcomes. Five principal determinants emerged with combined explanatory power of 83.64–88.16% variance, including population density (30.05–43.05% contribution), workforce concentration (30.69–33.83%), per capita fiscal expenditure, primary industry GDP share, and per capita secondary education infrastructure. The predominance of demographic and economic density factors underscored their critical role in shaping healthcare resource allocation patterns, necessitating priority consideration in policy optimization frameworks.

The analysis identified five structural determinants governing spatial healthcare disparities. Population density demonstrated polarization effects, with hyperdense city clusters exhibiting facility congestion while hypodense peripheries faced service scarcity and accessibility deficits (20, 76). Fiscal capacity, operationalized through per capita public expenditure, regulated healthcare investment magnitude, where increased allocations corresponded to infrastructure expansion, service optimization, and measurable outcome improvements including mortality reduction and longevity extension (77). Primary industry-dependent regions encountered compounded accessibility barriers from geographic isolation, structurally limiting advanced care availability while increasing reliance on primary care system to address occupation-environmental health risks (78, 79). Educational attainment mediated healthcare utilization efficiency through health literacy enhancement (80), establishing quantifiable associations between academic achievement and service optimization (81). Workforce concentration generated dual effects. It might exacerbate demand–supply imbalances through accelerated urbanization (82, 83) while paradoxically strengthen healthcare workforce retention in industrial clusters through economic attractiveness (84). These interdependent mechanisms collectively explained the structural foundations of healthcare inequities, supporting the implementation of differentiated allocation frameworks that addressed regional specificities through targeted policy interventions (85).

### 4.4 Regional policy implications across space and time

This study proposed a spatiotemporally informed policy framework to optimize healthcare equity through three governance strategies. First, the spatiotemporal evaluation framework could serve as a dynamic decision-support mechanism. The evaluation system would enable real-time identification of regions with equity deterioration through spatial Gini coefficient trends of composite scores, guide evidence-based resource reallocation via spatiotemporal hotspot and coldspot analysis, and further facilitate iterative policy adjustments through systematic monitoring of allocation outcomes. Second, the implementation of spatiotemporally adaptive governance protocols is highly recommended to address regional heterogeneities through differentiated interventions. For example, declining equity areas require prioritized establishment of cross-regional medical alliances to reduce access gaps, while physician-deficient areas necessitate incentive systems integrating service-credit portability, accelerated career pathways, and tax-benefit package to stabilize workforce allocation. Third, healthcare resource prioritization should be stratified according to economic structure and urbanization levels. Primary industry regions need infrastructure investments for basic care capacity enhancement and disease-specific competency building, while city healthcare development areas focus on specialized service networks and medical technology innovation to address advanced care demands. This framework bridges macroscale equity monitoring with context-specific implementation, offering a scalable model for precision health governance aligned with SDGs.

### 4.5 Interaction mechanisms and optimization strategies among multiple healthcare resource indicators

The proposed spatiotemporal evaluation framework highlights the critical role of multiple resource interplay in driving healthcare equity. A co-evolutionary analysis of hospital infrastructure and physician workforce reallocation reveals that hospitals unlock substantially greater allocation efficiencies when their capacity expansions are synchronized with targeted physician distribution adjustments, an effect most pronounced in rapidly urbanizing zones (86). These intertwined dynamics underscore the necessity of integrated policy design: hospital bed-and-facility growth must be coupled with robust physician incentive and mobility schemes to forestall local resource agglomeration and access gaps. The combined use of the composite index and its constituent indicators equips decision-makers with a dual-perspective diagnostic system to locate resource-complementary regions. In areas exhibiting bed abundance but physician scarcity, for instance, rotational clinical training programs and telemedicine platforms can be strategically introduced to optimize utilization and redress short-board effects (87).

Furthermore, the bidirectional feedback loop between composite and individual metrics refines precision governance across scales. The composite index charts macroscale allocation trajectories and, through spatial Gini coefficient trends, identifies priority intervention zones, whereas single indicators spotlight granular resource deficits demanding immediate operational attention (88). This dual-scale diagnostic architecture enables a stratified implementation pathway: high-scoring regions can concentrate on optimizing specialized service networks, while low-scoring areas should prioritize foundational infrastructure build-out. Crucially, monitoring the influence of single-indicator improvements (e.g., increases in physician density) on composite index dynamics creates an iterative, spatiotemporally adaptive mechanism for fine-tuning resource redistribution, thereby enhancing allocation accuracy and sustaining equity over time (89, 90).

### 4.6 Limitations

This study has limitations. First, the selected healthcare resource indicators were hospitals, hospital beds, and physicians. We did not include other indicators such as primary healthcare facilities, nursing staff, and medical equipment due do data unavailability, potentially constraining the granularity of multi-dimensional characterization. Second, the city-level analytical scale prevented granular analysis of intra-city resource distribution patterns across sub-city administrative units, thereby limiting insights into localized disparities in healthcare accessibility. Third, the current evaluation framework primarily relied on quantitative metrics for resource distribution, neglecting critical dimensions such as service quality assessments, facility upgrading indices, and patient accessibility metrics, which resulted in an incomplete examination of operational effectiveness. Fourth, the analysis did not incorporate cross-provincial healthcare utilization due to the absence of comprehensive interprovincial patient flow data and the jurisdictional basis of resource allocation, leaving spatiotemporal equity considerations only partially addressed. These methodological constraints underscore the need for future research to expand indicator systems, conduct multiscale spatial analyses, integrate quality-effectiveness and patient flow metrics, and accommodate cross-boundary utilization patterns to enhance the comprehensiveness and applicability of healthcare resource evaluations. Finally, it is worth noting that a common practice in current mainstream research, namely, first reducing multiple target variables into a single composite indicator, has significant drawbacks, as it loses crucial multi-target information. Future methodological studies on BSTVC should aim to jointly model the spatiotemporal nonstationarity of multiple targets within a unified framework (91, 92).

## 5 Conclusion

We innovatively propose a joint spatiotemporal evaluation framework to assess multiple healthcare resources across three key dimensions: equity, agglomeration, and driving factors. This approach reveals the limited synergy among different resource types and addresses a critical gap in existing research, which has largely overlooked the interconnected dynamics of multiple indicators. Meanwhile, this multi-dimensional joint spatiotemporal evaluation framework systematically reveals the evolving patterns of city healthcare resource allocation in China and offers integrated macro- and micro-level evidence to support informed policymaking. At macro level, despite quantitative growth in healthcare resources, equity improvements remain limited with pronounced spatial disparities and declining trends in certain regions. At micro level, the identified “short-board effects” and intra-regional variations highlight systemic coordination challenges in current resource allocation. Furthermore, we quantitatively identify key drivers and elucidate their heterogeneous spatiotemporal impacts on resource distribution, establishing a theoretical foundation for implementing cross-regional monitoring, gradient compensation policies, and interregional cooperation. The proposed framework would not only guide healthcare resource optimization and support SDG acceleration globally, but also offer methodological innovations for assessing resource allocation in education, transportation, ecological environments, and other public services.

## Data Availability

The raw data supporting the conclusions of this article will be made available by the authors, without undue reservation.

## Footnotes

^1^ https://data.cnki.net/

^2^ https://giovanni.gsfc.nasa.gov/giovanni/

^3^ https://zenodo.org/
