# Supplementary material for: Joint spatiotemporal evaluation of multiple healthcare resources: hospitals, hospital beds and physicians across 365 Chinese cities over 22 years
Source: Front Public Health. 2025 Sep 12;13:1642295. doi: 10.3389/fpubh.2025.1642295 (PMC12463904; doi:10.3389/fpubh.2025.1642295)
Supplement: Supplementary file 1 [file Supplementary_file_1.pdf]

Supplementary Material

1 Supplementary Tables

Supplementary Table S1 Socioeconomic and environmental factors of multiple healthcare resources across Chinese cities.

Type	No.	Factor name	Unit
Socioeconomic factors	F1	Population density	People/km²
	F2	Per capita gross national product	RMB
	F3	Per capita general fiscal revenue	RMB
	F4	Per capita general fiscal expenditure	RMB
	F5	Average wages of employees on the job	RMB
	F6	Per capita total retail sales of consumer goods	RMB
	F7	Factory enterprise density	Per km²
	F8	General primary school student density	People/km²
	F9	General secondary school student density	People/km²
	F10	Average number of schools per pupil	/
	F11	Total wages per employee on the job	RMB
	F12	Per capita year-end loan balance of financial institutions	RMB
	F13	Per capita collection of books in public libraries	/
	F14	Primary industry value-added/GDP ratio	%
	F15	Per capita secondary school teachers	People/people
	F16	Per capita secondary schools	/
	F17	Per capita quota above wholesale and retail trade gross merchandise sales	RMB
	F18	Employed population density	People/km²
	F19	Secondary industry value-added/GDP ratio	%
	F20	Per capita primary school teachers	People/people
	F21	Primary school teacher ERDI	/
	F22	Primary school ERDI	/
	F23	Secondary school ERDI	/
	F24	Secondary school teacher ERDI	/
	F25	Per capita household deposit balance	RMB
	F26	Per capita fixed asset investment	RMB
	F27	Number of mobile phone users per capita	/
	F28	Number of broadband users per capita	/
Environmental factors	F29	SO_2	μg/m³
	F30	PM_1	μg/m³
	F31	$PM_{2.5}$	μg/m³
	F32	PM_{10}	μg/m³
	F33	Nighttime light index	/
	F34	NDVI	/

Supplementary Table S2 Spatial Gini coefficient of multiple healthcare resources.

Province	Spatial Gini coefficient			
	Composite scores	Hospitals	Beds	Physicians
Ningxia	0.06	0.07	0.09	0.09
Tianjin	0.07	0.06	0.15	0.09
Jilin	0.08	0.06	0.29	0.2
Beijing	0.08	0.08	0.08	0.13
Guizhou	0.08	0.09	0.23	0.11
Hebei	0.08	0.08	0.27	0.16
Fujian	0.08	0.06	0.3	0.2
Hunan	0.08	0.08	0.26	0.28
Shanxi	0.09	0.1	0.16	0.22
Shandong	0.09	0.09	0.29	0.29
Liaoning	0.09	0.1	0.33	0.28
Heilongjiang	0.11	0.12	0.37	0.28
Jiangsu	0.11	0.09	0.42	0.25
Shanghai	0.11	0.09	0.41	0.29
Zhejiang	0.11	0.09	0.39	0.33
Shaanxi	0.11	0.1	0.24	0.27
Guangxi	0.12	0.05	0.44	0.4
Sichuan	0.12	0.1	0.38	0.49
Jiangxi	0.13	0.05	0.38	0.36
Gansu	0.13	0.18	0.39	0.42
Henan	0.13	0.11	0.33	0.39
Yunnan	0.15	0.16	0.48	0.32
Qinghai	0.16	0.17	0.21	0.27
Chongqing	0.17	0.17	0.32	0.29
Anhui	0.19	0.12	0.31	0.48
Tibet	0.21	0.25	0.18	0.2
Hubei	0.22	0.15	0.39	0.41
Guangdong	0.25	0.16	0.33	0.55
Hainan	0.3	0.2	0.51	0.21
Inner Mongolia	0.47	0.43	0.62	0.49
Xinjiang	0.58	0.29	0.62	0.79

Supplementary Table S3 Spatiotemporal hot and cold spots of multiple healthcare resources across Chinese cities.

Classifications	Number of hot and cold spots and percentage (%)			
	Composite scores	Hospitals	Beds	Physicians
Consecutive coldspot	32 (8.77)	8 (2.19)	82 (22.47)	29 (7.95)
Consecutive hotspot	24 (6.58)	18 (4.93)	1 (0.27)	37 (10.14)
Intensifying coldspot	40 (10.96)	53 (14.52)	/	/
Intensifying hotspot	41 (11.23)	28 (7.67)	43 (11.78)	19 (5.21)
New hotspot	6 (1.64)	5 (1.37)	1 (0.27)	9 (2.47)
Oscillating hotspot	1 (0.27)	/	/	/
Persistent hotspot	18 (4.93)	30 (8.22)	/	/
Persistent coldspot	/	14 (3.84)	/	/
Sporadic hotspot	/	18 (4.93)	/	3 (0.82)
Diminishing hotspot	/	6 (1.64)	/	/
Historical coldspot	/	1 (0.27)	/	/
No pattern detected	203 (55.62)	184 (50.41)	238 (65.21)	268 (73.43)

Supplementary Table S4 Socioeconomic and environmental factors' VIF value and their node purity indicator ranking.

Factor	VIF	Node purity indicator	Renumber
Population density	4.50	1.52	X1
NDVI	2.02	1.30	X10
Employed population density	3.19	0.90	X5
$PM_{2.5}$	1.88	0.81	X8
Nighttime light index	4.64	0.68	X9
Per capita general fiscal expenditure	3.93	0.50	X2
PM_1	2.16	0.37	X7
Per capita secondary schools	1.54	0.33	X4
Primary industry value-added/GDP ratio	3.63	0.33	X3
Per capita primary school teachers	1.65	0.28	X6
Per capita secondary school teachers	2.35	0.25	/
Primary school ERDI	3.06	0.22	/
Per capita gross national product	3.72	0.21	/
Per capita quota above wholesale and retail trade gross merchandise sales	2.80	0.20	/
Average wages of employees on the job	3.78	0.19	/
Secondary industry value-added/GDP ratio	2.22	0.19	/
Number of mobile phone users per capita	3.44	0.16	/
Average number of schools per pupil	3.86	0.14	/
Per capita collection of books in public libraries	3.06	0.13	/

2 Supplementary Text

Supplementary Text S1 The classifications of hotspot and coldspot.
Spatiotemporal hotspot classification follows standardized statistical criteria:

- *Consecutive Coldspot/Hotspot*: Significant clustering in ≥2 consecutive terminal periods, never previously observed (<90% historical significance)
- *Intensifying Coldspot/Hotspot*: Significant clustering in majority periods (including current) with progressively increasing intensity
- *New Hotspot*: Significant hotspot in current period without prior occurrence
- *Oscillating Hotspot*: Current significant hotspot preceded by significant coldspot (<90% hotspot persistence)
- *Persistent Coldspot/Hotspot*: >90% periods as statistically significant cluster without substantial intensity change
- *Sporadic Hotspot*: Current significant hotspot with intermittent historical occurrence (<90% persistence; never significant coldspot)
- *Diminishing Hotspot*: >90% periods as significant hotspot (including current) with progressively decreasing intensity
- *Historical Coldspot*: >90% periods as significant coldspot without current status
